# Genome-Wide Screens Identify Genes Responsible for Intrinsic Boric Acid Resistance in *Escherichia coli*

**DOI:** 10.1007/s12011-024-04129-0

**Published:** 2024-03-11

**Authors:** Bekir Çöl, Merve Sezer Kürkçü, Esra Di̇bek

**Affiliations:** 1 Faculty of Science, Department of Biology, Mugla Sitki Kocman University, Mugla, Turkey; 2 Research Laboratories Center, Biotechnology Research Center, Mugla Sitki Kocman University, Mugla, Turkey; 3 Research and Application Center For Research Laboratories, Mugla Sitki Kocman University, Mugla, Turkey; 4 Köyceğiz Vocational School of Health Services, Department of Pharmacy Services, Mugla Sitki Kocman University, Mugla, Turkey

**Keywords:** Boric acid, Genome-wide screen, *Escherichia coli*, Gene

## Abstract

Boric acid (BA) has antimicrobial properties and is used to combat bacterial infections, including Enterobacteria. However, the molecular mechanisms and cellular responses to BA are still unknown. This genomics study aims to provide new information on the genes and molecular mechanisms related to the antimicrobial effect of BA in *Escherichia coli*. The Keio collection of *E. coli* was used to screen 3985 single-gene knockout strains in order to identify mutant strains that were sensitive or hypersensitive to BA at certain concentrations. The mutant strains were exposed to different concentrations of BA ranging from 0 to 120 mM in LB media. Through genome-wide screens, 92 mutants were identified that were relatively sensitive to BA at least at one concentration tested. The related biological processes in the particular cellular system were listed. This study demonstrates that intrinsic BA resistance is the result of various mechanisms acting together. Additionally, we identified eighteen out of ninety-two mutant strains (Delta_*aceF*, *aroK*, *cheZ*, *dinJ*, *galS*, *garP*, *glxK*, *nohA*, *talB*, *torR*, *trmU*, *trpR*, *yddE*, *yfeS*, *ygaV*, *ylaC*, *yoaC*, *yohN*) that exhibited sensitivity using other methods. To increase sensitivity to BA, we constructed double and triple knockout mutants of the selected sensitive mutants. In certain instances, engineered double and triple mutants exhibited significantly amplified effects. Overall, our analysis of these findings offers further understanding of the mechanisms behind BA toxicity and intrinsic resistance in *E. coli*.

## Introduction

The molecular mechanisms underlying boron toxicity, as well as the physiological causes and cellular processes involved, are not yet well understood. Boron toxicity has been linked to the chemical properties of boron, which tightly binds to the cis-diol groups of certain biomolecules, including ribose, Ado-Met, and NADH [1, 2]. Boron requirement and toxicity are intriguing because although some living organisms require or benefit from a small amount of boron, it becomes toxic above a certain level [3, 4]. Therefore, in many parts of the world where the boron level is high, the risk of boron toxicity for living organisms is a concern [5]. Boron is also used as a food preservative, insecticide, and treatment for vaginal diseases caused by Saccharomyces and Candida [6, 7]. Boron has significant effects on a variety of organisms. One of these effects is its role as a micronutrient in plant growth, as demonstrated by Warrington in 1923 [8]. Furthermore, boron is vital for plant structure and cell walls, making it an essential element [9]. It has also been reported to be essential for some species of Cyanobacteria and *Bacillus boroniphilus* [10, 11].

Studies have shown that boron may contribute to promoting certain metabolic activities, as indicated by various investigations [12–14]. Several important studies conducted with yeast have demonstrated that a membrane protein actively transports boron [15]. Furthermore, boron has been shown to be necessary for certain animals and single-cell eukaryotes [16, 17]. The essentiality of boron for certain organisms has been increasingly considered over time. The study found that plants with mutations in NIP5:1 are more susceptible to boron deficiency in root and branch development due to the lack of boron uptake [18]. Furthermore, it has been suggested that neutral boric acid [B(OH)3] can passively diffuse in addition to facilitating acid transport into the cell through the NIP5:1 protein concentration gradient along the plasma membrane [19]. Although there have been discussions on the existence of regulatory mechanisms in bacteria regarding intracellular boron levels, no experimental studies have been conducted yet. Only a few suggestions have been made regarding the variation of intracellular boron levels among different species [20]. To date, no genes or proteins related to boron resistance or efflux functions have been identified in the bacterial world.

Bacteria adapt to environmental changes or extreme conditions through the regulation of their genes, proteins, and metabolites. Understanding the dynamics of this regulatory network is a key goal of biology. *Escherichia coli* is a commonly used model organism in microbiological studies and is one of the most well-characterized organisms in this field [21]. The K12 strains *E. coli* MG1655 [22] and *E. coli* W3110 [23] have been extensively used for genomic studies. We utilized the Keio mutant collection, an important example of new generation genomic research in *E. coli* [24]. This mutant collection can be used to study the effects of any chemical or agent that can kill a bacterium at a certain dose. In this study, we screened the Keio mutant line to identify the genes conferring intrinsic resistance to boric acid (BA) in *E. coli*. The roles of some genes in intrinsic BA resistance were discovered and discussed.

## Materials and Methods

### Escherichia coli Strains and Plasmids

The Keio mutant line, derived from the starting *E. coli* strain BW25113 [25], was used in this study [24]. For complementation purposes, pCA24N plasmids containing the target genes were employed [26]. An empty pCA24N plasmid, which contains a chloramphenicol resistance (Cm^R^), was used as a control. The open reading frame (ORF) regions cloned into this vector are transcribed by the T5-*lac* promoter, which is inducible by isopropyl-β-D-thiogalactoside (IPTG) [26]. The plasmids and strains used in this study are listed in Table 1.

**Table 1:** The strains and plasmids used in this study

Strains and plasmids	Genotype	Reference
Strains		
*E. coli* K-12 BW25113	*lacI*^*q*^* rrnB*_*T14*_* ΔlacZ*_*WJ16*_* hsdR514 ΔaraBAD*_*AH33*_* ΔrhaBAD*_*LD78*_	[25]
*ΔygaV*	BW25113 *ΔygaV* ΩKm^R^	
*ΔtorR*	BW25113 *ΔtorR* ΩKm^R^	
*ΔyfeS*	BW25113 *ΔyfeS* ΩKm^R^	
*ΔyoaC*	BW25113 *ΔyoaC* ΩKm^R^	
*ΔdinJ*	BW25113* ΔdinJ* ΩKm^R^	
*ΔylaC*	BW25113* ΔylaC* ΩKm^R^	
*ΔgarP*	BW25113* ΔgarP* ΩKm^R^	
*ΔyohN*(*rcnB*)	BW25113* ΔyohN(rcnB)* ΩKm^R^	
*ΔglxK*	BW25113* ΔglxK* ΩKm^R^	[24]
*ΔnohA*	BW25113* ΔnohA* ΩKm^R^	
*ΔyddE*	BW25113* ΔyddE* ΩKm^R^	
*ΔtrpR*	BW25113* ΔtrpR* ΩKm^R^	
*ΔaceF*	BW25113* ΔaceF* ΩKm^R^	
*ΔgalS*	BW25113* ΔgalS* ΩKm^R^	
*ΔtalB*	BW25113* ΔtalB* ΩKm^R^	
*ΔtrmU*	BW25113* ΔtrmU* ΩKm^R^	
*ΔcheZ*	BW25113* ΔcheZ* ΩKm^R^	
*E. coli* K-12 BW25113 (pCA24N)	*lacI*^*q*^* rrnB*_*T14*_* ΔlacZ*_*WJ16*_* hsdR514 ΔaraBAD*_*AH33*_* ΔrhaBAD*_*LD78*_*Cm*^*R*^*; lacI*^*q*^*, pCA24N*	This study
*ΔygaV* (pCA24N::*ygaV*)	*ΔygaV* ΩKm^R^ pCA24N P_T5-lac_:: *ygaV*^+^	
*ΔtorR* (pCA24N::*torR*)	*ΔtorR* ΩKm^R^ pCA24N P_T5-lac_::*torR*^+^	
*ΔyfeS* (pCA24N::*yfeS*)	*ΔyfeS* ΩKm^R^ pCA24N P_T5-lac_::*yfeS*^+^	
*ΔyoaC* (pCA24N::*yoaC*)	*ΔyoaC* ΩKm^R^ pCA24N P_T5-lac_::*yoaC*^+^	
*ΔdinJ* (pCA24N::*dinJ*)	*ΔdinJ* ΩKm^R^ pCA24N P_T5-lac_::*dinJ*^+^	
*ΔylaC* (pCA24N::*ylaC*)	*ΔylaC* ΩKm^R^ pCA24N P_T5-lac_::*ylaC*^+^	
*ΔgarP* (pCA24N::*garP*)	*ΔgarP* ΩKm^R^ pCA24N P_T5-lac_::*garP*^+^	
*ΔyohN* (pCA24N::*yohN*)	*ΔyohN* ΩKm^R^ pCA24N P_T5-lac_::*yohN*^+^	
*ΔglxK* (pCA24N::*glxK*)	*ΔglxK* ΩKm^R^ pCA24N P_T5-lac_::*glxK*^+^	This study
*ΔnohA* (pCA24N::*nohA*)	*ΔnohA* ΩKm^R^ pCA24N P_T5-lac_::*nohA*^+^	
*ΔyddE* (pCA24N::*yddE*)	*ΔyddE* ΩKm^R^ pCA24N P_T5-lac_::*yddE*^+^	
*ΔtrpR* (pCA24N::*trpR*)	*ΔtrpR* ΩKm^R^ pCA24N P_T5-lac_::*trpR*^+^	
*ΔaceF* (pCA24N::*aceF*)	*ΔaceF* ΩKm^R^ pCA24N P_T5-lac_::*aceF*^+^	
*ΔgalS* (pCA24N::*galS*)	*ΔgalS* ΩKm^R^ pCA24N P_T5-lac_::*galS*^+^	
*ΔtalB* (pCA24N::*talB*)	*ΔtalB* ΩKm^R^ pCA24N P_T5-lac_::*talB*^+^	
*ΔtrmU* (pCA24N::*trmU*)	*ΔtrmU* ΩKm^R^ pCA24N P_T5-lac_::*trmU*^+^	
*ΔcheZ* (pCA24N::*cheZ*)	*ΔcheZ* ΩKm^R^ pCA24N P_T5-lac_::*cheZ*^+^	
*ΔygaV* (pCA24N)	*Δyga*V ΩKm^R^ pCA24N	
*ΔtorR* (pCA24N)	*ΔtorR* ΩKm^R^ pCA24N	
*E. coli* K-12		
BW25113 *ΔyfeS* (pCA24N)	*ΔyfeS* ΩKm^R^ pCA24N	This study
BW25113 *ΔyoaC* (pCA24N)	*ΔyoaC* ΩKm^R^ pCA24N	
BW25113 *ΔdinJ* (pCA24N)	*ΔdinJ* ΩKm^R^ pCA24N	
BW25113 *ΔylaC* (pCA24N)	*ΔylaC* ΩKm^R^ pCA24N	
BW25113 *ΔgarP* (pCA24N)	*ΔgarP* ΩKm^R^ pCA24N	
BW25113 *ΔyohN*(*rcnB*) (pCA24N)	*ΔyohN(rcnB)* ΩKm^R^ pCA24N	
BW25113 *ΔglxK* (pCA24N)	*ΔglxK* ΩKm^R^ pCA24N	
BW25113 *ΔnohA* (pCA24N)	*ΔnohA* ΩKm^R^ pCA24N	
BW25113 *ΔyddE* (pCA24N)	*ΔyddE* ΩKm^R^ pCA24N	
BW25113 *ΔtrpR* (pCA24N)	*ΔtrpR* ΩKm^R^ pCA24N	
BW25113 *ΔaceF* (pCA24N)	*ΔaceF* ΩKm^R^ pCA24N	
BW25113 *ΔgalS* (pCA24N)	*ΔgalS* ΩKm^R^ pCA24N	
BW25113 *ΔtalB* (pCA24N)	*ΔtalB* ΩKm^R^ pCA24N	
BW25113 *ΔtrmU* (pCA24N)	*ΔtrmU* ΩKm^R^ pCA24N	
BW25113 *ΔcheZ* (pCA24N)	*ΔcheZ* ΩKm^R^ pCA24N	
Plasmids		
pCA24N	pCA24N; Cm^R^; *lacI*^*q*^	
pCA24N::*ygaV*	pCA24N P_T5-lac_::*ygaV*^+^; Cm^R^; *lacI*^*q*^	
pCA24N::*torR*	pCA24N P_T5-lac_::*torR*^+^; Cm^R^; *lacI*^*q*^	[26]
pCA24N::*yfeS*	pCA24N P_T5-lac_::*yfeS*^+^; Cm^R^; *lacI*^*q*^	
pCA24N::*yoaC*	pCA24N P_T5-lac_::*yoaC*^+^; Cm^R^; *lacI*^*q*^	
pCA24N::*dinJ*	pCA24N P_T5-lac_::*dinJ*^+^;Cm^R^; *lacI*^*q*^	
pCA24N::*ylaC*	pCA24N P_T5-lac_::*ylaC*^+^;Cm^R^; *lacI*^*q*^	
pCA24N::*garP*	pCA24N P_T5-lac_::*garP*^+^;Cm^R^; *lacI*^*q*^	[26]
pCA24N::*yohN(rcnB)*	pCA24N P_T5-lac_::*yohN(rcnB)*^+^;Cm^R^; *lacI*^*q*^	
pCA24N::*glxK*	pCA24N P_T5-lac_::*glxK*^+^;Cm^R^; *lacI*^*q*^	
pCA24N:: *nohA*	pCA24N P_T5-lac_::*nohA*^+^;Cm^R^; *lacI*^*q*^	
pCA24N:: *yddE*	pCA24N P_T5-lac_::*yddE*^+^;Cm^R^; *lacI*^*q*^	
pCA24N:: *trpR*	pCA24N P_T5-lac_::*trpR*^+^;Cm^R^; *lacI*^*q*^	
pCA24N:: *aceF*	pCA24N P_T5-lac_::*aceF*^+^;Cm^R^; *lacI*^*q*^	
pCA24N:: *galS*	pCA24N P_T5-lac_::*galS*^+^;Cm^R^; *lacI*^*q*^	
pCA24N:: *talB*	pCA24N P_T5-lac_::*talB*^+^;Cm^R^; *lacI*^*q*^	
pCA24N:: *trmU*	pCA24N P_T5-lac_::*trmU*^+^;Cm^R^; *lacI*^*q*^	
pCA24N:: *cheZ*	pCA24N P_T5-lac_::*cheZ*^+^;Cm^R^; *lacI*^*q*^	

### Genome-Wide Screening of Intrinsic BA Resistance-Associated Genes with the Replicator

The Keio mutant strains are stored in 96 deep well plates with 15% glycerol at − 80 °C. They were then transferred to 96 deep well plates containing 1 ml of Luria Bertani (LB) medium per well using a cryoreplicator consisting of 96 pins. The plates were incubated at 37 °C. Next, all strains were transferred with the replicator to deep well plates containing 50 μg/ml of kanamycin in LB medium. After incubation for 3 h, the strains were plated on LB agar medium with varying BA concentrations (0, 25, 50, 80, 100, 120 mM). The mutant strains were then incubated for 3 days to grow.

### Sequential Spotting

The mutant strains that were identified as sensitive to BA during screening studies underwent a sequential spot test. A single colony was inoculated into LB agar containing 50 μg/ml kanamycin without BA from the mutant strain’s stock. After confirming colony purity, the mutants were grown in LB at 37 °C until they reached a density of OD_600_ 0.5. They were then serially diluted (1/2, 1/4, 1/8, and 1/16). The reproductive status of the mutants was assessed by spotting 5-μl cultures onto LB agar medium with varying concentrations of BA. As the concentration of BA increased, it became toxic to the bacterium, leading to reduced bacterial growth. The plates were incubated for 72 h at 37 °C, and bacterial growth was recorded in tabular form.

### Determination of MIC

To determine the minimum inhibitory concentration (MIC) of the mutants [27], single colony cultures were grown on LB agar. The mutants were then inoculated onto LB agar medium containing 50 μg/ml kanamycin. After overnight growth, a colony was selected and inoculated into 5 ml of LB liquid medium with kanamycin (50 μg/ml) and left to incubate overnight at 37 °C. Sterile microplates were used to aliquot LB broth medium containing 50 μg/ml kanamycin and different concentrations of BA. The mutant strains, which were sensitive, were inoculated into a broth medium with an initial OD_600_ value of 0.05 and incubated at 37 °C. Daily monitoring was conducted to observe growth, and the MIC value was determined for each strain.

### Complementation of Sensitive Mutant Strains

To obtain a sufficient concentration of the recombinant plasmid vector containing the target gene (pCA24N::target gene), *E. coli* strains carrying the plasmids were cultivated and then the recombinant vectors carrying the target gene were isolated using a plasmid isolation kit (GeneAll 101–150). Then, the recombinant plasmid was transformed using the heat shock method into the particular mutant (*E. coli* BW25113::Δ*gene*) [28]. The susceptibility of the cells that regained the gene was reassessed by growing them in LB broth with varying BA concentrations, 100 μM IPTG, and 25 μg/ml chloramphenicol. The assessment was conducted using the MIC test described above.

### BA Sensitivity Analysis of Double and Triple Mutants

In the BW25113 background, we generated double and triple mutants via P1 transduction [29]. The aim of the study was to observe the mutants and determine if their susceptibility to BA is increased by the knockout of identified genes. The protocols published by Silhavy et al. (1984) were followed [30]. To generate a double mutant, the kanamycin gene cassette was deleted through FLP recombination using plasmid pCP20. Kanamycin-resistant (Km^R^) transductants were obtained after overnight incubation at 37 °C. We obtained double and triple mutants and confirmed the absence of the target genes using PCR.

### Data Mining, Analysis, and Interpretation

The activities of knockout genes in susceptible mutant strains were investigated using the EcoCyc at http://ecocyc.org/ [31] and UniProt databases, and the corresponding UniProt IDs of the proteins were obtained (https://www.uniprot.org/). The knockout genes of sensitive mutant strains were classified according to their systems and subsystems using the EcoCyc Database and Omics Dashboard (Pathway Tools, https://ecocyc.org/dashboard/dashboard-intro.shtml?orgid=ECOLI) [31]. Enrichment of the selected gene hits was carried out using the DAVID database (https://david.ncifcrf.gov/tools.jsp) [32, 33] with an FDR of 0.1 and a cut-off score of 2 SD from the mean. The STRING database (https://string-db.org/) [34] was also used to explore the direct or indirect relationships between the knockout genes of the identified susceptible mutant strains.

## Results

### Genome-Wide Screening of the Mutants for BA Sensitivity

**Fig. 1:**
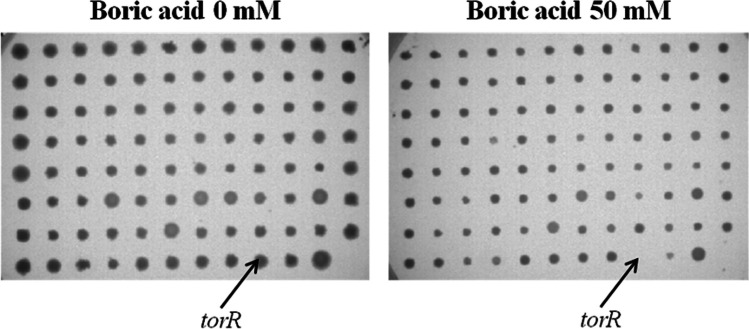
Effect of 50 mM BA on K-12 BW25113Δ*torR* ΩKm^R^ mutant *E. coli* strain. LB plates without any BA (left panel) and LB plates with 50 mM BA (right panel) were used to culture 96 mutants from the plate in Keio collection. Among them, *ΔtorR* mutant failed to grow on the plate with 50 mM BA. Further investigation on pure colonies confirmed its hypersensitivity

A total of 3985 mutants from the Keio collection were screened in triplicate across six different BA concentrations, generating an initial dataset of nearly 71,730 data points (23,910 × 3). An example from one microplate is shown in Fig. 1. The mutants that showed reduced or no growth at specific BA concentrations were recorded. Out of 3985 mutants, 92 sensitive mutants were determined after comparing them to the wild type and surrounding mutants. The table presents gene information and the concentrations of BA (in mM) that were tested for the strains. The dark gray color indicates good growth, while the color scale that fades towards white represents a decrease in growth, with white indicating no growth. “Wt” denotes the control strain (wild type), while the other strains are the sensitive mutants identified by their 4-letter gene names in Table 2.

**Table 2:**
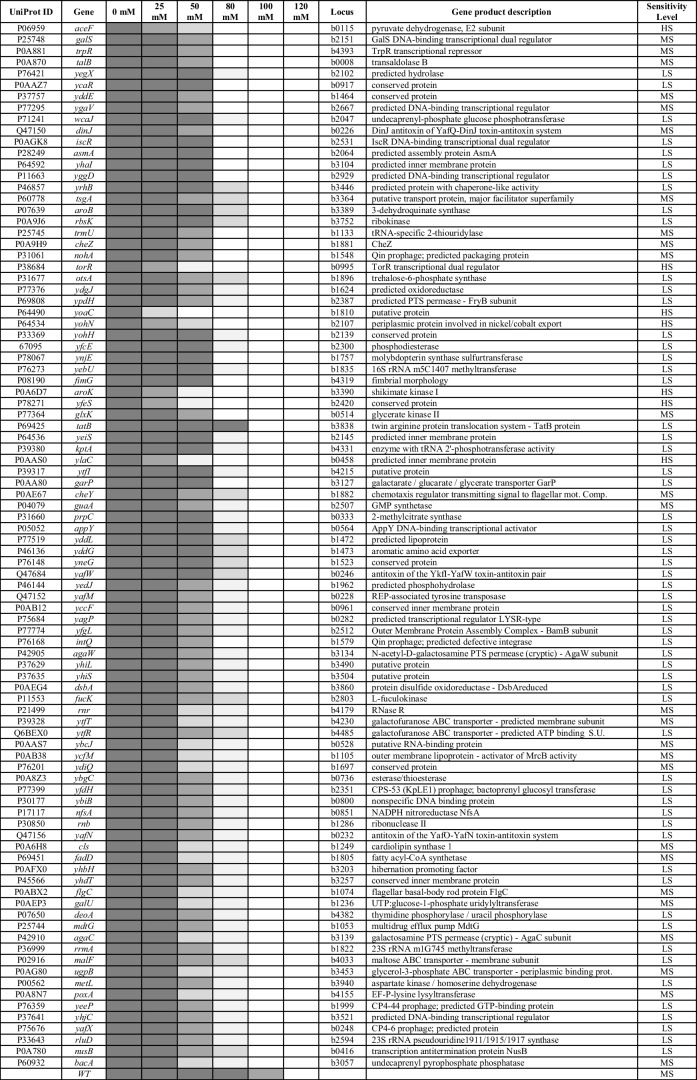
List of the 92 mutants sensitive to BA based on genome-wide screens*

The mutants, identified through screening studies, were categorized into three sensitivities: highly sensitive (HS), moderately sensitive (MS), and low sensitive (LS). The mutants’ responses to different concentrations of BA determined their sensitivity levels. Mutants that ceased reproduction or exhibited limited growth at 25 mM were classified as highly sensitive (HS), at 50 mM as moderately sensitive (MS), and at 80 mM as low sensitive (LS) (Table 2).

The study found that the absence of certain genes caused the BA-sensitive phenotype. A decrease in colony size or lack of reproduction of mutants indicates BA sensitivity. The study identified 92 genes associated with BA sensitivity (Table 2). These genes were classified based on their activity using Pathway Tools and the EcoCyc databases. It was observed that one gene was involved in more than one system or subsystem (Table 3).

**Table 3:** BA-sensitive gene hits organized in system and subsystem

System	Subsystem	Gene ID	Gene	Product	Sensitivity level
Biosynthesis	Amino acid biosynthesis	EG10590	*metL*	Fused aspartate kinase/homoserine dehydrogenase 2	LS
		EG10074	*aroB*	3-dehydroquinate synthase	LS
		EG10081	*aroK*	Shikimate kinase 1	HS
	Carbohydrate biosynthesis	G7098	*wcaJ*	UDP-glucose:undecaprenyl-P glucose-1-P transferase	LS
		EG11319	*galU*	UTP-glucose-1-P uridylyltransferase	MS
		EG11751	*otsA*	Trehalose 6-P synthase	LS
	Cofactor, carrier, and vitamin biosynthesis	EG11297	*dsbA*	Thiol:disulfide oxidoreductase – DsbA oxidized	LS
		EG10590	*metL*	Fused aspartate kinase/homoserine dehydrogenase 2	LS
		G6952	*ynjE*	Molybdopterin synthase sulfurtransferase	LS
		EG11665	*bacA*	Undecaprenyl pyrophosphate phosphatase	MS
	Fatty acid and lipid biosynthesis	EG11608	*clsA*	Cardiolipin synthase A	MS
	Metabolic regulator biosynthesis	EG11751	*otsA*	Trehalose 6-P synthase	LS
	Nucleoside and nucleotide biosynthesis	EG10420	*guaA*	GMP synthetase	MS
		EG10219	*deoA*	Thymidine phosphorylase	LS
	Other biosynthesis	EG11297	*dsbA*	Thiol:disulfide oxidoreductase—DsbAoxidized	LS
		EG10590	*metL*	Fused aspartate kinase/homoserine dehydrogenase 2	LS
		EG10074	*aroB*	3-dehydroquinate synthase	LS
		EG10081	*aroK*	Shikimate kinase 1	HS
Degradation	Amino acid degradation	EG10420	*guaA*	GMP synthetase	MS
	Carbohydrates and carboxylates degradation	EG10818	*rbsK*	Ribokinase	LS
		G6283	*glxK*	Glycerate 2-kinase 2	MS
		G6198	*prpC*	2-methylcitrate synthase	LS
		EG10025	*aceF*	AceF-dihydrolipoate	HS
		EG11530	*fadD*	Long-chain fattyacid-CoA ligase	MS
		EG10350	*fucK*	L-fuculokinase	LS
		EG11319	*galU*	UTP-glucose-1-P uridylyltransferase	MS
	Fatty acid and lipid degradation	EG11530	*fadD*	Long-chain fattyacid-CoA ligase	MS
	Nucleoside and nucleotide degradation	EG10219	*deoA*	Thymidine phosphorylase	LS
	Secondary metabolite degradation	G6283	*glxK*	Glycerate-kinase 2	MS
Energy		EG11556	*talB*	Transaldolase B	MS
		EG11162	*yggD*	Fumarase E	LS
Other pathways	Enzymes not in pathways	EG11261	*nfsA*	NADPH-dependent nitro/quinone reductase NfsA	LS
		EG12098	*rluD*	23S rRNA pseudouridine1911/1915/1917 synthase	LS
		G7008	*yebU*	16S rRNA m5C1407 methyltransferase	LS
		EG10151	*cheZ*	CheZ protein for chemotaxis	MS
		EG11259	*rnr*	RNase R	LS
		EG12207	*rrmA*	23S rRNA m1G745 methyltransferase	LS
		EG11211	*poxA*	EF-P-lysine lysyltransferase	MS
		G7220	*yfdH*	Bactoprenol glucosyl transferase CPS-53 (KpLE1) prophage	LS
	Inorganic nutrient metabolism	EG10420	*guaA*	GMP synthetase	MS
	Macromolecule modification	EG11297	*dsbA*	Thiol:disulfide oxidoreductase—DsbAoxidized	LS
		EG11344	*trmU*	tRNA-specific 2-thiouridylase	MS
		EG11620	*rnb*	RNase II	LS
Response to stimulus	Detoxification proteins	EG11343	*mdtG*	Efflux pump MdtG	LS
	Other proteins involved in stimulus response	EG10150	*cheY*	CheY-acetylated	MS
		EG10314	*fimG*	FimG fimbriae minor subunit type 1	LS
		EG11262	*tsgA*	Putative transporter TsgA	MS
		EG10151	*cheZ*	CheZ protein for chemotaxis	MS
		EG12615	*torR*	DNA binding transcriptional dual regulator TorR	HS
		EG11530	*fadD*	Long-chain-fatty-acid–CoA ligase	MS
		G359	*flgC*	FlgC flagellar basal body protein	LS
	Proteins involved in response to cold	EG11259	*rnr*	RNase R	LS
		EG11751	*otsA*	Trehalose 6-P synthase	LS
	Proteins involved in response to DNA damage	EG10219	*deoA*	Thymidine phosphorylase	LS
		G6116	*yafN*	Antitoxin YafN	LS
		G6920	*ydiQ*	Putative electron transfer flavoprotein subunit YdiQ	MS
		EG10555	*malF*	Maltose ABC transporter membrane subunit MalF	LS
		G7139	*yohN*	Periplasmic protein for nickel/cobalt export	HS
		EG11751	*otsA*	Trehalose 6-P synthase	LS
	Proteins involved in response to osmotic stress	EG11751	*otsA*	Trehalose 6-P synthase	LS
	Proteins involved in response to starvation	EG10050	*appY*	DNA binding transcriptional activator AppY, DLP12 prophage	LS
		EG11047	*ugpB*	sn-glycerol 3-P ABC transporter periplasmic binding protein	MS
	Proteins involved in response to pH	EG11211	*poxA*	EF-P-lysine lysyltransferase	MS
Central dogma	DNA metabolism	G6112	*yafM*	REP-associated tyrosine transposase	LS
		G6116	*yafN*	Antitoxin YafN	LS
	Protein metabolism	EG10150	*cheY*	CheY-acetylated	MS
		EG11681	*yhbH*	Ribosome hibernation-promoting factor	LS
		EG10151	*cheZ*	CheZ protein for chemotaxis	MS
		EG12879	*ybcJ*	Putative RNA-binding protein YbcJ	MS
		EG11211	*poxA*	EF-P-lysine lysyltransferase	MS
	RNA metabolism	G7397	*ygaV*	Putative DNA binding transcriptional regulator YgaV	MS
		G7928	*kptA*	RNA 2′-phosphotransferase	LS
		G6110	*dinJ*	Antitoxin/DNA binding transcriptional repressor DinJ	MS
		EG12098	*rluD*	23S rRNA pseudouridine1911/1915/1917 synthase	LS
		G7008	*yebU*	16S rRNA m5C1407 methyltransferase	LS
		EG11259	*rnr*	RNase R	LS
		EG12615	*torR*	DNA binding transcriptional dual regulator TorR	HS
		EG11344	*trmU*	tRNA-specific 2-thiouridylase	MS
		EG10050	*appY*	DNA binding transcriptional activator, AppY DLP12 prophage	LS
		EG11029	*trpR*	DNA binding transcriptional repressor TrpR	MS
		EG12207	*rrmA*	23S rRNA m1G745 methyltransferase	LS
		G6116	*yafN*	Antitoxin YafN	LS
		EG11620	*rnb*	RNase II	LS
		EG10666	*nusB*	Transcription antitermination protein	LS
		EG11211	*poxA*	EF-P-lysine lysyltransferase	MS
		EG12247	*yhjC*	Putative DNA binding transcriptional regulator YhjC	LS
		EG10365	*galS*	DNA binding transcriptional dual regulator GalS	MS
		G7326	*iscR*	DNA binding transcriptional dual regulator IscR	LS
	Transcription proteins	G6110	*dinJ*	Antitoxin/DNA binding transcriptional repressor DinJ	MS
		EG10666	*nusB*	Transcription antitermination protein	LS
		EG12247	*yhjC*	Putative DNA binding transcriptional regulator YhjC	LS
	Translation proteins	EG12879	*ybcJ*	Putative RNA-binding protein YbcJ	MS
		EG11211	*poxA*	EF-P-lysine lysyltransferase	MS
Virulence-related	Proteins involved in locomotion	EG10150	*cheY*	CheY-acetylated	MS
	Proteins involved in biofilm formation	EG10314	*fimG*	FimG fimbriae minor subunit type 1	LS
	Proteins involved in cell adhesion (except biofilm)	EG10314	*fimG*	FimG fimbriae minor subunit type 1	LS
	Proteins involved in response to antibiotic	EG11297	*dsbA*	Thiol:disulfide oxidoreductase DsbAoxidized	LS
		EG12020	*yohH*	mdtQ	LS
	Proteins involved in biofilm formation	G6110	*dinJ*	Antitoxin/DNA binding transcriptional repressor DinJ	MS
	Proteins involved in response to antibiotic	EG11343	*mdtG*	Efflux pump MdtG	LS
	Proteins involved in locomotion	EG10151	*cheZ*	CheZ protein for chemotaxis	MS
		G359	*flgC*	FlgC flagellar basal body protein	LS
	Proteins involved in response to antibiotic	EG11665	*bacA*	Undecaprenyl pyrophosphate phosphatase	MS
		EG10081	*aroK*	Shikimate kinase 1	HS
Genes not present in any subsystem		G6868	*ydgJ*	Putative oxidoreductase YdgJ	LS
		EG12710	*yedJ*	Putative HD superfamily phosphohydrolase YedJ	LS
		EG11825	*yddE*	PF02567 family protein YddE	MS
		G7250	*ypdH*	Putative PTS enzyme IIB component	LS
		G7192	*yfcE*	Phosphodiesterase YfcE	LS
		G6153	*yagP*	Putative LysR family protein	LS
		EG12238	*yhiS*	yhiS	LS
		G7870	*ytfI*	Protein YtfI	LS
		G7079	*yeeP*	yeeP	LS
		G6809	*yneG*	DUF4186 domain-containing protein	LS
		G7134	*yegX*	Putative glycosyl hydrolase YegX	LS
Regulation	Sigma factor regulons	EG10150	*cheY*	CheY-acetylated	MS
		EG10314	*fimG*	FimG fimbriae minor subunit type 1	LS
		G7633	*agaW*	N-acetyl-D-galactosamine specific PTS truncated enzyme IIC component	LS
		EG11297	*dsbA*	Thiol:disulfide oxidoreductase—DsbAoxidized	LS
		G7320	*yfgL*	Outer membrane protein assembly factor BamB	LS
		EG11634	*nohA*	Putative prophage DNA-packaging protein NohA	MS
		G6837	*intQ*	Putative defective integrase, Qin prophage;	LS
		G6110	*dinJ*	Antitoxin/DNA binding transcriptional repressor DinJ	MS
		EG10818	*rbsK*	Ribokinase	LS
		EG11681	*yhbH*	Ribosome hibernation-promoting factor	LS
		EG11261	*nfsA*	NADPH-dependent nitro/quinone reductase NfsA	LS
		EG10590	*metL*	Fused aspartate kinase/homoserine dehydrogenase 2	LS
		EG10420	*guaA*	GMP synthetase	MS
		G7808	*tatB*	Twin arginine protein translocation system	LS
		EG10151	*cheZ*	CheZ protein for chemotaxis	MS
		G6283	*glxK*	Glycerate 2-kinase 2	MS
		EG12713	*yddG*	Amino acid exporter YddG	LS
		G6994	*yoaC*	DUF1889 domain-containing protein YoaC	HS
		G6121	*yafW*	Antitoxin of the YkfI-YafW toxin-antitoxin pair	LS
		EG12615	*torR*	DNA binding transcriptional dual regulator TorR	HS
		G6198	*prpC*	2-methylcitrate synthase	LS
		EG10050	*appY*	DNA binding transcriptional activator AppY, DLP12 prophage	LS
		EG11029	*trpR*	DNA binding transcriptional repressor TrpR	MS
		EG10025	*aceF*	AceF-dihydrolipoate	HS
		EG12760	*garP*	Galactarate/D-glucarate transporter GarP	LS
		G6123	*yafX*	CP4-6 prophage; protein YafX	LS
		EG11580	*ybiB*	Nonspecific DNA binding protein YbiB	LS
		EG11530	*fadD*	Long-chain fattyacid -CoA ligase	MS
		EG11047	*ugpB*	sn-glycerol 3-P ABC transporter periplasmic binding protein	MS
		EG10219	*deoA*	Thymidine phosphorylase	LS
		EG11665	*bacA*	Undecaprenyl pyrophosphate phosphatase	MS
		G6116	*yafN*	Antitoxin YafN	LS
		G359	*flgC*	FlgC flagellar basal body protein	LS
		EG10350	*fucK*	L-fuculokinase	LS
Regulation *continued	Sigma factor regulons *continued	EG10666	*nusB*	Transcription antitermination protein NusB	LS
		EG11620	*rnb*	RNase II	LS
		EG12770	*agaC*	Galactosamine-specific PTS enzyme IIC component	MS
		G7693	*yhdT*	DUF997 domain-containing protein YhdT	LS
		EG10074	*aroB*	Dehydroquinate synthase	LS
		EG10081	*aroK*	Shikimate kinase 1	HS
		G7261	*yfeS*	PF05406 family protein YfeS	HS
		EG10555	*malF*	Maltose ABC transporter membrane subunit MalF	LS
		EG12247	*yhjC*	Putative DNA binding transcriptional regulator YhjC	LS
		G7139	*yohN*	Periplasmic protein for nickel/cobalt export	HS
		EG11751	*otsA*	Trehalose 6-P synthase	LS
		EG10365	*galS*	DNA binding transcriptional dual regulator GalS	MS
		G7326	*iscR*	DNA binding transcriptional dual regulator IscR	LS
		EG12227	*yhiL*	yhiL	LS
		G6472	*ycaR*	PF03966 family protein YcaR	LS
	Signal transduction pathways	EG10150	*cheY*	CheY-acetylated	MS
		EG12615	*torR*	DNA binding transcriptional dual regulator TorR	HS
	Transcription factors	G6110	*dinJ*	Antitoxin/DNA binding transcriptional repressor DinJ	MS
		EG12615	*torR*	DNA binding transcriptional dual regulator TorR	HS
		EG10050	*appY*	DNA binding transcriptional activator AppY DLP12 prophage	LS
		EG11029	*trpR*	DNA binding transcriptional repressor TrpR	MS
		EG10365	*galS*	DNA binding transcriptional dual regulator GalS	MS
		G7326	*iscR*	DNA binding transcriptional dual regulator IscR	LS
Regulation *continued	Transcription factor regulons	EG10150	*cheY*	CheY-acetylated	MS
		EG10314	*fimG*	FimG fimbriae minor subunit type 1	LS
		G7633	*agaW*	N-acetyl-D-galactosamine specific PTS truncated enzyme IIC comp	LS
		EG12518	*ytfR*	Galactofuranose ABC transporter putative ATP binding subunit	LS
		EG11297	*dsbA*	Thiol:disulfide oxidoreductase—DsbAoxidized	LS
		EG11634	*nohA*	Putative prophage DNA-packaging protein	MS
		G7763	*yrhB*	Putative heat shock chaperone	LS
		G6837	*intQ*	Putative defective integrase, Qin prophage	LS
		G7397	*ygaV*	Putative DNA binding transcriptional regulator	MS
		G6110	*dinJ*	Antitoxin/DNA binding transcriptional repressor	MS
		EG10818	*rbsK*	Ribokinase	LS
		EG11262	*tsgA*	Putative transporter TsgA	MS
		EG11261	*nfsA*	NADPH-dependent nitro/quinone reductase NfsA	LS
		EG10590	*metL*	Fused aspartate kinase/homoserine dehydrogenase2	LS
		EG11681	*yhbH*	Ribosome hibernation-promoting factor	LS
		EG11343	*mdtG*	Efflux pump MdtG	LS
		EG10420	*guaA*	GMP synthetase	MS
		G7008	*yebU*	16S rRNA m5C1407 methyltransferase	LS
		EG10151	*cheZ*	CheZ protein for chemotaxis	MS
		G6283	*glxK*	Glycerate 2 kinase	MS
		EG12713	*yddG*	Amino acid exporter YddG	LS
		EG11259	*rnr*	RNase R	LS
		G6994	*yoaC*	DUF1889 domain-containing protein YoaC	HS
		EG12615	*torR*	DNA binding transcriptional dual regulator TorR	HS
		EG11344	*trmU*	tRNA-specific 2-thiouridylase	MS
		G6198	*prpC*	2-methylcitrate synthase	LS
		EG10050	*appY*	DNA binding transcriptional activator, DLP12 prophage	LS
		EG11029	*trpR*	DNA binding transcriptional repressor TrpR	MS
		G7618	*yhaI*	Putative inner membrane protein	LS
		EG10025	*aceF*	AceF-dihydrolipoate	HS
		EG12760	*garP*	Galactarate/D-glucarate transporter GarP	LS
		EG11530	*fadD*	Long-chain-fatty-acid–CoA ligase	MS
		EG10219	*deoA*	Thymidine phosphorylase	LS
		EG11580	*ybiB*	Nonspecific DNA binding protein YbiB	LS
		EG11047	*ugpB*	sn-glycerol 3-P ABC transporter periplasmic binding protein	MS
		G359	*flgC*	FlgC flagellar basal body protein	LS
		G6116	*yafN*	Antitoxin YafN	LS
		EG11665	*bacA*	Undecaprenyl pyrophosphate phosphatase	MS
		EG11110	*ybgC*	Esterase/thioesterase	LS
		EG10350	*fucK*	L-fuculokinase	LS
		EG10666	*nusB*	Transcription antitermination protein NusB	LS
		EG12770	*agaC*	Galactosamine-specific PTS enzyme IIC component	MS
		EG10074	*aroB*	3-dehydroquinate synthase	LS
		EG10081	*aroK*	Shikimate kinase 1	HS
		G7261	*yfeS*	PF05406 family protein YfeS	HS
		EG10555	*malF*	Maltose ABC transporter membrane subunit	LS
		EG12247	*yhjC*	Putative DNA binding transcriptional regulator	LS
		EG12520	*ytfT*	Galactose ABC transporter membrane subunit putative	MS
		G7139	*yohN*	Periplasmic protein for nickel/cobalt export	HS
		EG10365	*galS*	DNA binding transcriptional dual regulator	MS
		G7326	*iscR*	DNA binding transcriptional dual regulator	LS
Cell exterior	Cell wall biogenesis/organization proteins	G6565	*ycfM*	Outer membrane lipoprotein -act of MrcB act	MS
		EG11665	*bacA*	Undecaprenyl pyrophosphate phosphatase	MS
	Flagellar proteins	EG10150	*cheY*	CheY-acetylated	MS
		EG10151	*cheZ*	Chemotaxis protein CheZ	MS
		G359	*flgC*	FlgC flagellar basal body protein	LS
	Lipopolysaccharide metabolism proteins	G7098	*wcaJ*	UDP-glucose-1-P transferase	LS
		EG11319	*galU*	UTP-glucose-1-P uridylyltransferase	MS
	Outer membrane proteins	G7320	*yfgL*	Outer membrane protein assembly factor	LS
		EG12020	*yohH*	mdtQ	LS
		G6565	*ycfM*	Outer membrane lipoprotein—act of MrcB act	MS
		G6773	*yddL*	Putative uncharacterized protein YddL	LS
	Periplasmic proteins	EG11297	*dsbA*	Thiol:disulfide oxidoreductase -DsbAoxidized	LS
		EG11361	*asmA*	Putative assembly protein AsmA	LS
		G6952	*ynjE*	Molybdopterin synthase sulfurtransferase	LS
		EG11047	*ugpB*	sn-glycerol 3-P ABC transporter periplasmic binding	MS
		G359	*flgC*	FlgC flagellar basal body protein	LS
		G7139	*yohN*	Periplasmic protein for nickel/cobalt export	HS
	Pilus proteins	EG10314	*fimG*	FimG fimbriae minor subunit type 1	LS
	Plasma membrane proteins	EG12518	*ytfR*	Galactose ABC transporter ATP binding subunit putative	LS
		EG11262	*tsgA*	Putative transporter TsgA	MS
		EG11343	*mdtG*	Efflux pump MdtG	LS
		G7808	*tatB*	Twin arginine protein translocation system	LS
		EG10151	*cheZ*	Chemotaxis protein CheZ	MS
		G7098	*wcaJ*	UDP-glucose-1-P transferase	LS
		EG12713	*yddG*	Amino acid exporter YddG	LS
		EG11361	*asmA*	Putative assembly protein AsmA	LS
		G6496	*yccF*	PF03733 family inner membrane protein	LS
		G7144	*yeiS*	DUF2542 domain-containing protein YeiS	LS
		G7618	*yhaI*	Putative inner membrane protein	LS
		G6253	*ylaC*	Putative inner membrane protein	HS
		EG12760	*garP*	Galactarate/D-glucarate transporter GarP	LS
		EG11047	*ugpB*	sn-glycerol 3-P ABC transporter periplasmic binding	MS
		EG11530	*fadD*	Long-chain-fatty-acid–CoA ligase	MS
		EG11665	*bacA*	Undecaprenyl pyrophosphate phosphatase	MS
		EG11110	*ybgC*	Esterase/thioesterase	LS
		G7693	*yhdT*	DUF997 domain-containing protein YhdT	LS
		EG12770	*agaC*	Galactosamine-specific PTS enzyme IIC component	MS
		EG10555	*malF*	Maltose ABC transporter membrane subunit	LS
		G7220	*yfdH*	Bactoprenol glucosyl transferase CPS-53 (KpLE1) prophage	LS
		EG11608	*clsA*	Cardiolipin synthase A	MS
		EG12520	*ytfT*	Galactose ABC transporter membrane subunit putative	MS
	Transport proteins	EG12518	*ytfR*	Galactofuranose ABC transporter putative ATP binding subunit	LS
		EG11343	*mdtG*	Efflux pump MdtG	LS
		G7808	*tatB*	Twin arginine protein translocation system -	LS
		EG12713	*yddG*	Amino acid exporter YddG	LS
		EG12760	*garP*	Galactarate/D-glucarate transporter GarP	LS
		EG11047	*ugpB*	sn-glycerol 3-P ABC transporter periplasmic binding protein	MS
		EG10555	*malF*	Maltose ABC transporter membrane subunit	LS
		EG12520	*ytfT*	Galactofuranose ABC transporter putative membrane subunit	MS

### Assessment of BA Sensitivity and Resistance Using Sequential Spotting and MIC Tests

**Fig. 2:**
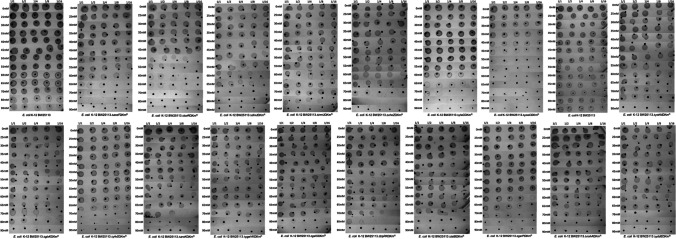
Spot tests to determine the BA sensitivity of the 18 mutant strains. The mutants were subjected to sequential spotting under different concentrations of BA, as described in the “Materials and Methods” section. Five different dilutions of the bacterial culture (1/1, 1/2, 1/4, 1/8, 1/16) and 13 different BA concentrations were used. The growth of the mutants and the wild-type strain is illustrated, with the particular strain name listed below each picture

Ninety-two mutants underwent spot tests to determine their sensitivity to BA compared to the wild-type strain. The results confirmed that 18 mutants, namely Δ*aceF*, Δt*orR*, Δ*dinJ*, Δ*trmU*, Δ*cheZ*, Δ*ylaC*, Δ*yoaC*, Δ*yohN*, Δ*glxK*, Δ*yfeS*, Δ*aroK*, Δ*ygaV*, Δ*galS*, Δ*trpR*, Δ*talB*, Δ*garP*, Δ*nohA*, and Δ*yddE*, exhibited relatively higher sensitivity (Fig. 2).

MIC (minimum inhibitory concentration) values were determined using the microtube dilution method to compare the resistance levels of the mutant strains in solid and liquid media. The MIC test for the mutants was conducted in LB broth medium containing various BA concentrations (0, 25, 30, 40, 45, 50, 60, 65, 70, 80, 90, 100 mM). The reproductive status was monitored daily. Table 4 presents the MIC values of the 18 sensitive mutants to BA and the control strain. The control strain had a MIC value of 100 mM BA, while the mutants had reduced MIC values ranging from 40 to 80 mM BA, confirming their sensitivity or hypersensitivity (Table 4).

**Table 4:** Boric acid MIC values (mM) of 18 sensitive mutants

Mutant strain	Mutant strain genotype (K-12 BW25113 Δgene mutant)	MIC value BA (mM)	Mutant strain	Mutant strain genotype (K-12 BW25113 Δgene mutant)	MIC value BA (mM)
*aceF*	Δ*aceF* ΩKm^R^	80	*glxK*	Δ*glxK*ΩKm^R^	80
*torR*	Δ*torR* ΩKm^R^	60	*aroK*	Δ*aroK* ΩKm^R^	80
*yfeS*	Δ*yfeS* ΩKm^R^	50	*ygaV*	Δ*ygaV* ΩKm^R^	70
*yoaC*	Δ*yoaC* ΩKm^R^	40	*galS*	Δ*galS* ΩKm^R^	70
*dinJ*	Δ*dinJ* ΩKm^R^	65	*trpR*	Δ*trpR* ΩKm^R^	80
*ylaC*	Δ*ylaC* ΩKm^R^	80	*talB*	Δ*talB* ΩKm^R^	90
*garP*	Δ*garP* ΩKm^R^	70	*nohA*	Δ*nohA* ΩKm^R^	90
*yohN*	Δ*yohN(rcnB)* ΩKm^R^	80	*yddE*	Δ*yddE* ΩKm^R^	80
*trmU*	Δ*trmU* ΩKm^R^	70	*cheZ*	Δ*cheZ* ΩKm^R^	80
*BW25113*	K-12 BW25113	100			

The growth inhibition of 18 susceptible mutants to BA was demonstrated using the MIC test. The test was conducted in LB broth medium supplemented with 50 μg/ml kanamycin and varying concentrations of BA (0, 25, 30, 40, 45, 50, 60, 65, 70, 80, 90, 100 mM). The control strain, E. coli BW25113 (wt), was employed

### Complementation of BA-Sensitive Mutant Strains

To conduct the complementation experiments, we transformed the target gene into the corresponding mutants listed in Table 1 using the recombinant plasmid. For each mutant, we also prepared controls consisting of cells containing the empty plasmid pCA24N without any gene insert. We assessed the sensitivity to BA using the MIC test and monitored reproductive status daily. The complementation experiments revealed that the mutants *ΔglxK*, *ΔcheZ*, *ΔtalB*, *ΔgalS*, and *ΔnohA* did not exhibit complementation. However, it was demonstrated that the mutants *ΔtrmU*, *ΔaceF*, *ΔtorR*, *ΔdinJ*, *ΔylaC*, *ΔyoaC*, *ΔyohN*, *ΔyfeS*, *ΔygaV*, *ΔtrpR*, *ΔgarP*, and *ΔyddE* regained their genes and resistance levels to BA. This confirms that the BA-sensitive phenotype of the mutant is attributed to the specific gene of interest (Table 5).

**Table 5:** MIC values of BA (mM) after complementation of the mutants with plasmid-encoded gene

Strains (*E. coli* K-12 BW25113 wt or Δgene mutant)	MIC value BA (mM)	
BW25113 (pCA24N) (wild type)	100	Control strain
Δ*ygaV* (pCA24N)	80	Before complementation
Δ*ygaV* (pCA24N::*ygaV*)	100	After complementation
Δ*torR* (pCA24N)	80	Before complementation
Δ*torR* (pCA24N::*torR*)	100	After complementation
Δ*yfeS* (pCA24N)	45	Before complementation
Δ*yfeS* (pCA24N::*yfeS*)	65	After complementation
Δ*yoaC* (pCA24N)	45	Before complementation
Δ*yoaC* (pCA24N::*yoaC*)	60	After complementation
Δ*dinJ* (pCA24N)	45	Before complementation
Δ*dinJ* (pCA24N::*dinJ*)	90	After complementation
Δ*ylaC* (pCA24N)	90	Before complementation
Δ*ylaC* (pCA24N::*ylaC*)	100	After complementation
Δ*garP* (pCA24N)	80	Before complementation
Δ*garP* (pCA24N::*garP*)	100	After complementation
Δ*yohN*(*rcnB*) (pCA24N)	80	Before complementation
Δ*yohN*(*rcnB*) (pCA24N::*yohN*(*rcnB*))	100	After complementation
Δ*glxK* (pCA24N)	90	Before complementation
Δ*glxK* (pCA24N::*glxK*)	90	After complementation
Δ*aceF* (pCA24N)	70	Before complementation
Δ*aceF* (pCA24N::*aceF*)	90	After complementation
Δ*trmU* (pCA24N)	60	Before complementation
Δ*trmU* (pCA24N::*trmU*)	80	After complementation
Δ*cheZ* (pCA24N)	80	Before complementation
Δ*cheZ* (pCA24N::*cheZ*)	80	After complementation
Δ*galS* (pCA24N)	70	Before complementation
Δ*galS* (pCA24N::*galS*)	90	After complementation
Δ*trpR* (pCA24N)	80	Before complementation
Δ*trpR* (pCA24N::*trpR*)	90	After complementation
Δ*nohA* (pCA24N)	90	Before complementation
Δ*nohA* (pCA24N::*nohA*)	90	After complementation
Δ*yddE* (pCA24N)	90	Before complementation
Δ*yddE* (pCA24N::*yddE*)	100	After complementation

Demonstration of growth inhibition by MIC test as a result of complementation experiments of selected sensitive mutants susceptible to BA. As a control, BW25113 containing pCA24N plasmid was used

### Testing the Sensitivity of Double and Triple Knockout Mutants to BA

Double and triple mutants were generated using P1 transductions. The mutants were assessed for their sensitivity to BA, and it was observed that some mutants displayed more pronounced effects. The results are presented in Table 6. The double and triple mutants obtained exhibited significantly higher sensitivity compared to the individual single mutants. For example, the *garP*-*ylaC* and *yoaC*-*yfeS* double mutants demonstrated a sensitivity of approximately 50 mM, while each parent mutant exhibited a sensitivity ranging from 50 to 80 mM. Moreover, the generated triple mutants displayed even greater sensitivity, reaching down to 25 mM BA, compared to the individual mutants, as observed in the cases of *ΔyoaCgarPylaC* and *ΔgarPyoaCyfeS*. These results confirm the BA sensitivity of the specific mutants. Additionally, we suggest that these sensitive triple mutants can be employed in other genomic library selection studies, using other BA-tolerant bacteria, to identify more candidate genes related to the effect of BA on bacteria.

**Table 6:** MIC values of single, double, and triple *E. coli* knockout mutants to boric acid

Strain	MIC (BA, mM)	Double mutant	MIC (BA, mM)	Triple mutant	MIC (BA, mM)
BW25113	100				
*ΔylaC*	80	*ΔgarP ylaC*	60	*ΔylaC garP yoaC*	25
*ΔyoaC*	45	*ΔyoaC yfeS*	40	*ΔylaC garP yfeS*	55
*ΔgarP*	80			*ΔyfeS yoaC garP*	25
*ΔyfeS*	45				

## Discussion

### BA-Sensitive Mutants of E. coli Were Identified Through Genome-Wide Screens, Spot, and MIC Tests

Boron is beneficial to living organisms in small amounts but can become toxic beyond a certain threshold. Additionally, boron exhibits antibacterial activity, although the molecular mechanisms behind this are unknown. Several publications have reported on the function of genes related to boron resistance and transport in yeast and plants [15, 35–37]. However, the molecular mechanisms underlying boron resistance or sensitivity in other organisms and bacteria remain unclear.

Advancements in genomics and transcriptomics have facilitated the utilization of functional genomic-based approaches to comprehend the global metabolic changes resulting from genotypic and/or environmental variations [38–42]. Our study employed a functional genomics approach to investigate the impact of BA on *E. coli*. A total of 3985 mutants were screened for sensitivity in the presence of increasing levels of BA.

The objective of this study was to investigate the genes and mechanisms responsible for intrinsic BA resistance in *E. coli*, a model bacterium. To achieve this, the Keio mutant line, consisting of single mutants of nonessential genes belonging to *E. coli*, was used [24]. The genome-wide screening of the mutants identified 92 knockout strains that showed increased susceptibility to BA, revealing genes involved in intrinsic BA resistance (Table 2). In addition, sequential spot tests and MIC experiments were conducted on these 92 mutants to determine their sensitivity to BA. The results showed that 18 of the mutants were particularly sensitive under all the methods tested (Fig. 2). The mutants’ sensitivity to BA ranged from most hypersensitive to sensitive in the following order: *ΔyoaC* > *ΔtorR* > *ΔglxK* > *ΔylaC* > *ΔaroK* > *ΔyfeS* > *ΔaceF* > *ΔdinJ* > *ΔcheZ* > *ΔtrmU* > *ΔygaV* > *ΔyohN* > *ΔgalS* > *ΔtalB* > *ΔtrpR* > *ΔyddE* > *ΔnohA* > *ΔgarP*. These mutants were found to be sensitive to BA following genome-wide screening, sequential spot, and MIC experiments.

### Complementation Studies and Generation of Triple Mutants Confirmed the BA-Sensitive Phenotype of the Particular Mutants

Complementation experiments were performed to investigate whether the sensitivity to BA was due to the knockout gene. The mutant strains, along with proper control strains, were subject to BA spot tests after transformation with the corresponding gene cloned into an expression plasmid. It was demonstrated that the mutants *ΔtrmU*, *ΔaceF*, *ΔtorR*, *ΔdinJ*, *ΔylaC*, *ΔyoaC*, *ΔyohN*, *ΔyfeS*, *ΔygaV*, *ΔtrpR*, *ΔgarP*, and *ΔyddE* were complemented with their respective genes (Table 5). This confirms that the BA-sensitive phenotype of each mutant is due to the deleted gene. Further studies with these mutants are recommended.

To elaborate on the particular genes’ effect on intrinsic BA resistance, we generated double and triple knockouts to test for increased BA sensitivity. We created double mutants of *ΔgarPylaC* and *ΔyoaCyfeS*, and from these, we generated three triple mutants: *ΔylaCgarPyoaC*, *ΔylaCgarPyfeS*, and *ΔyfeSyoaCgarP* (Table 6). The mutants with the *yoaC* knockout exhibited the most sensitive phenotype to BA. The single, double, and triple mutants, where *yoaC* is deleted, had BA sensitivity levels of 45 mM, 40 mM, and 25 mM, respectively. YoaC is particularly noteworthy for its effect on intrinsic BA resistance in *E. coli*. Further research is advised on this gene with respect to BA response. There is no information available about *yoaC* in the literature, and the function of this gene is unknown. Investigating the involvement of YoaC in BA stress could be a good starting point to elucidate the physiological function of YoaC in *E. coli* and other bacteria.

### Pathway and Network Analyses of the Genes of the Sensitive Mutants Provide Insights into Intrinsic Resistance to BA

It is important to note that different culturing conditions may yield different results, which could lead to the addition or omission of some genes specifically related to the studied process. With these limitations in mind, we discuss the corresponding genes in BA-sensitive mutants in terms of the affected systems. Our focus is primarily on the systems expected to exhibit high sensitivity, along with any other significant findings and noteworthy genes that did not display a response. To gain a broader perspective, we analyzed all 92 genes using Omics Dashboard and String approaches, with particular attention paid to the 18 genes in detail.

**Fig. 3:**
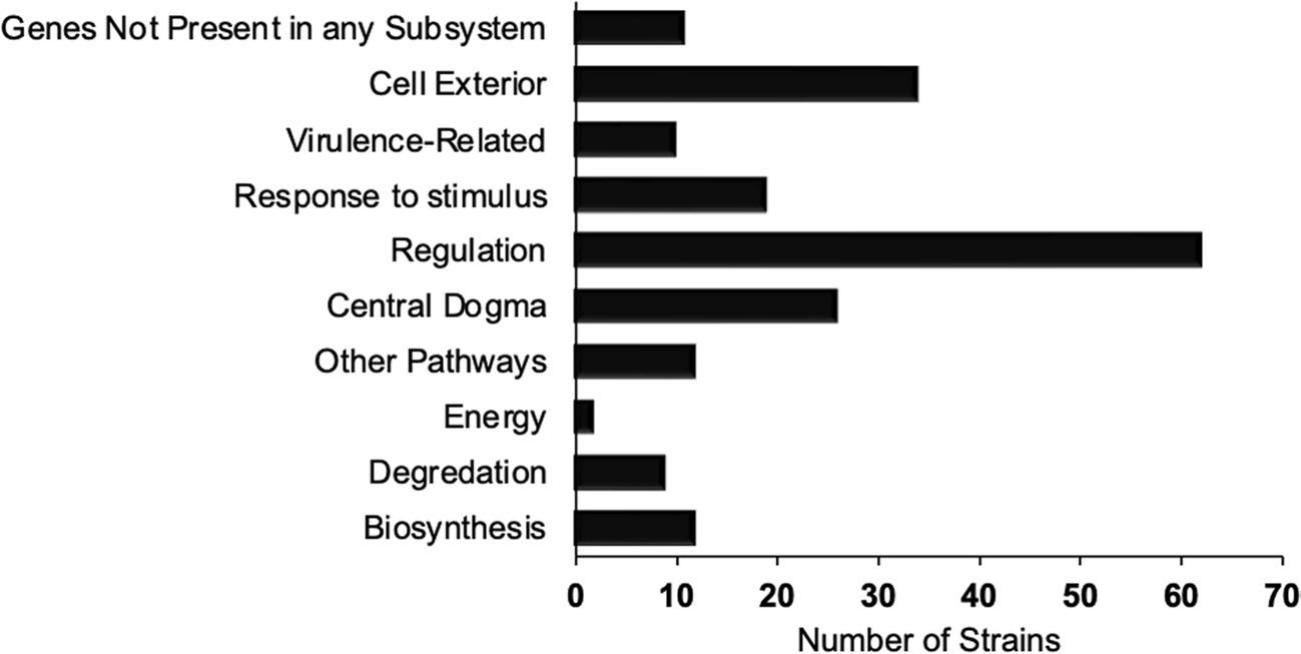
Strain numbers of subsystems. Each gene hit, which is associated with a BA-sensitive mutant strain, is mapped to specific cellular processes. It is important to note that gene hits may be associated with more than one process. The gene list was evaluated using Omics Dashboard (Pathway Tools) and the EcoCyc databases

Using Pathway Tools and the EcoCyc database, we mapped several gene hits to multiple systems and subsystems (Table 3 and Fig. 3). Our findings suggest that various cellular mechanisms, along with the corresponding physiological responses of *E. coli*, are involved in BA toxicity and resistance. This conclusion is supported by the identification of genes across multiple cellular systems, as shown in Table 3.

Out of the 92 BA-sensitive gene hits, a significant number were assigned to various cellular processes, including “Regulation,” such as sigma factor regulons, signal transduction pathways, transcription factors, and transcription factor regulons. Hits were observed in various categories, including “Cell exterior,” “Cell Wall Biogenesis-Organization Proteins,” “Flagellar Proteins,” “Lipopolysaccharide Metabolism Proteins,” “Outer Membrane Proteins,” “Periplasmic Proteins,” “Pilus Proteins,” “Plasma Membrane Proteins,” and “Transport Proteins.” Additionally, hits were found in the “Central dogma” category, which covers DNA Metabolism, Protein Metabolism, RNA Metabolism, Transcription Proteins, and Translation Proteins. The “Response to stimulus” category includes hits related to Detoxification Proteins, Other Proteins involved in Stimulus Response, Proteins Involved in Response to Cold, Proteins Involved in Response to DNA Damage, Proteins Involved in Response to Osmotic Stress, Proteins Involved in Response to Starvation, Proteins Involved in Response to pH, and others.

Overall, we observed 62, 34, 26, and 19 sensitive hits in the categories of “Regulation,” “Cell exterior,” “Central dogma,” and “Response to stimulus,” respectively (Fig. 3). These findings suggest that BA has an impact on several cellular processes to varying degrees.

**Fig. 4:**
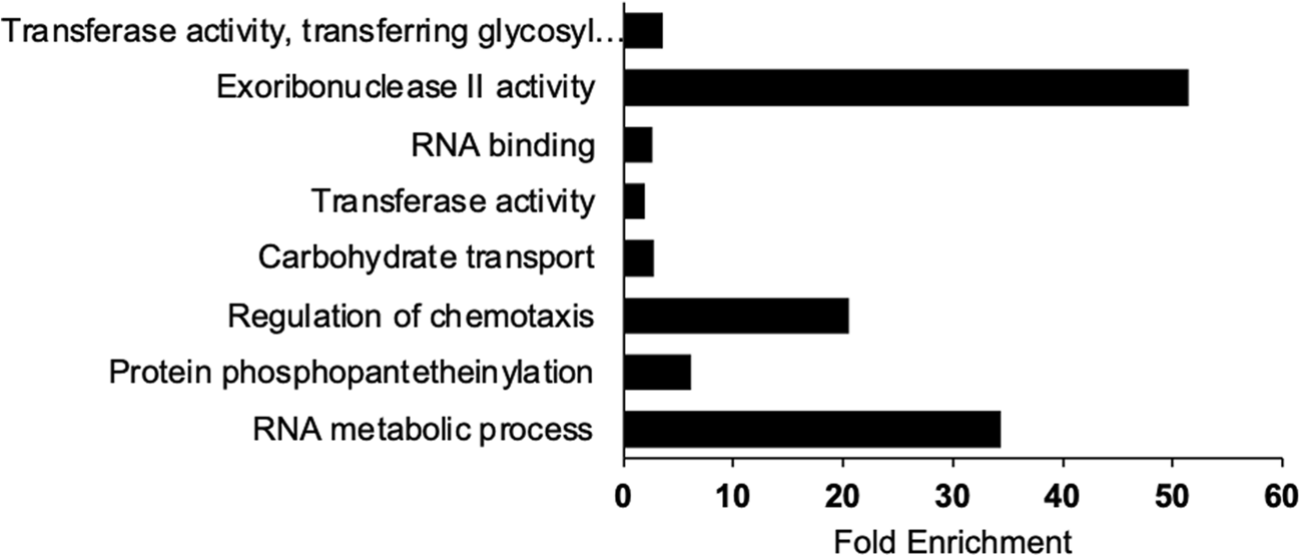
Enrichment of the corresponding genes in BA-sensitive mutants g. Clusters with three or more genes were considered (*p* value < 0.05). The DAVID gene functional classification (version 6.8) database was employed to assess the enrichment using *E. coli* K-12 genome. Only the particular processes with three or more genes were included

Enrichment analysis was performed on the 92 BA-sensitive gene hits using the DAVID pathway database. The analysis revealed that proteins related to exoribonuclease II activity were the most significantly affected among the BA-sensitive gene mutants, with an enrichment score of over 50. Additionally, highly affected protein groups include those associated with RNA metabolism and the regulation of chemotaxis, with fold enrichment values above 30 and 20, respectively (Fig. 4). Mutants lacking genes in RNA processing exhibit sensitivity to BA, suggesting that BA may target the processing and maturation of tRNAs and possibly other RNAs. Further research is suggested to investigate the effect of BA on bacteria in relation to RNA.

#### “Central Dogma and Cell Exterior Proteins”

Twenty-six mutants, classified in the “Central Dogma,” exhibited sensitivity to BA (Table 3). For instance, *trmU* (*mnmA*) is a gene involved in RNA metabolism. TrmU catalyzes the formation of the 2-thiouridine modification [43, 44] in the nucleoside 5-methylamino-methyl-2-thiourridine (mnm5s2U34) found in tRNA^Gln^, tRNA^Lys^, and tRNA^Glu^. These modifications regulate selective mRNA translation and have an impact on protein expression. Several studies have identified factors involved in mRNA splicing processes. One such study found that BA causes a reversible inhibition effect in pre-mRNA splicing [45], suggesting that BA interacts with RNA in the cell.

Our study identified 34 genes associated with the “Cell exterior” system (Fig. 2). One of these genes, YlaC, is an inner membrane protein (UniProt P0AASO) [46] that has been found to be highly sensitive to BA. Another gene, the *yohN* (*rcnB*) gene, encodes a protein involved in the nickel cobalt transport system [47, 48]. Additionally, a study showed that mutant strains lacking the *rcnB* gene were more susceptible to LL-37 and Magainin 2 antimicrobial peptides (AMPs) than the wild type [49]. The reason for the increased sensitivity of the *rcnB* mutant to BA remains unknown and requires further investigation.

The GarP protein, which belongs to the MFS (Major Facilitator Superfamily) transporter family, is involved in the transport of galactarate, glucarate, and glycerate [50, 51]. Biologically, it is classified within the scope of “anion and transmembrane transport.” GarP (UniProt P0AA80) is located in the inner membrane of the cell and contains 12 transmembrane domains. A study investigated the genes required for the intrinsic multidrug resistance of *E. coli* and identified *garP* as one of the genes involved. The study demonstrated that the *garP* mutant is more susceptible to chloramphenicol than the control strain [52], indicating a relationship between *garP* and antibiotic resistance. Our findings regarding the BA sensitivity of the *garP* mutant provide new information to the existing literature.

#### “Degradation”

Among the nine genes classified under degradation, the *aceF* gene appears to be the most sensitive. AceF is a part of the pyruvate dehydrogenase complex and is located in the cytosol. The pyruvate dehydrogenase enzyme is in charge of oxidatively decarboxylating pyruvate to produce acetyl-CoA. The AceF protein is specifically involved in the core region (E1) of the pyruvate dehydrogenase multi-enzyme complex and interacts with the 24-subunit [53, 54]. The deletion of *aceF* may have led to a reduction in energy production, potentially impacting the cells’ response to BA.

The *glxK* gene encodes glycerate kinase activity, which is involved in respiration. The product of this reaction was initially identified as 3-phosphoglycerate [55]. GlxK catalyzes the phosphorylation of D-glycerate and is involved in glycerolipid, amino acid, and glucose metabolism. The potential relationship between BA and glycerate could be a starting point for further investigations.

#### “Regulation”

The regulation category consists of 62 genes, including the *torR* mutant, which encodes a transcriptional regulator and belongs to the TorS/TorR two-component regulatory system. Initially, *torR* was found to regulate trimethylamine N-oxide reductase genes [56]. Another study discovered that TorR proteins accumulate at the poles of older cells and require interaction with DnaK and MreB proteins. This regulation is cell cycle-dependent and regulates the expression of many genes [57]. The TorS/TorR binary system has been linked to *E. coli*’s response to alkaline stress [58]. Furthermore, the *torR* gene has been shown to contribute to phosphomycin tolerance in *E. coli* (EHEC) O157:H7 and K12 strains [56, 59]. Therefore, there is a correlation between TorR activity and the response to antibacterial compounds and stress. It is worth noting that the *torR* mutant of *E. coli* is sensitive to BA.

YfeS is a conserved protein with an unknown function. The *yfeS* and *yfeK* genes are organized in the same operon and are controlled by the sigma 24 promoter (*yfeKp* sigma 24). Sigma 24 (*rpoE*, Sigma E factor) is involved in the expression of genes that encode membrane and periplasmic proteins in response to heat shock and other stresses. Sigma E triggers the transcription of many genes associated with heat shock and unfolded proteins. Heat shock also induces Sigma E’s expression [60]. Additionally, Sigma E is involved in resistance to zinc, cadmium, and copper [61–63]. Therefore, the association of Sigma E with stress and its binding site in the promoter of the *yfeS* gene can serve as an initial clue for investigating the relationship between BA stress and *yfeS*.

The *galS* gene encodes a protein with DNA-binding transcriptional dual regulatory activity. GalS, also known as the galactose isorepressor, is a DNA-binding transcription factor that represses the transcription of operons involved in the transport and catabolism of D-galactose [64–68]. Further research is needed to investigate any potential link between galactose and BA. TrpR, also known as a tryptophan transcriptional repressor, negatively regulates *trp* expression [69, 70]. The TrpR regulon is involved in tryptophan biosynthesis, transport, and regulation [71–74]. BA may be effective in tryptophan synthesis and potentially other aromatic amino acids, which requires further investigation in the future.

#### “Virulence Related”

**Fig. 5:**
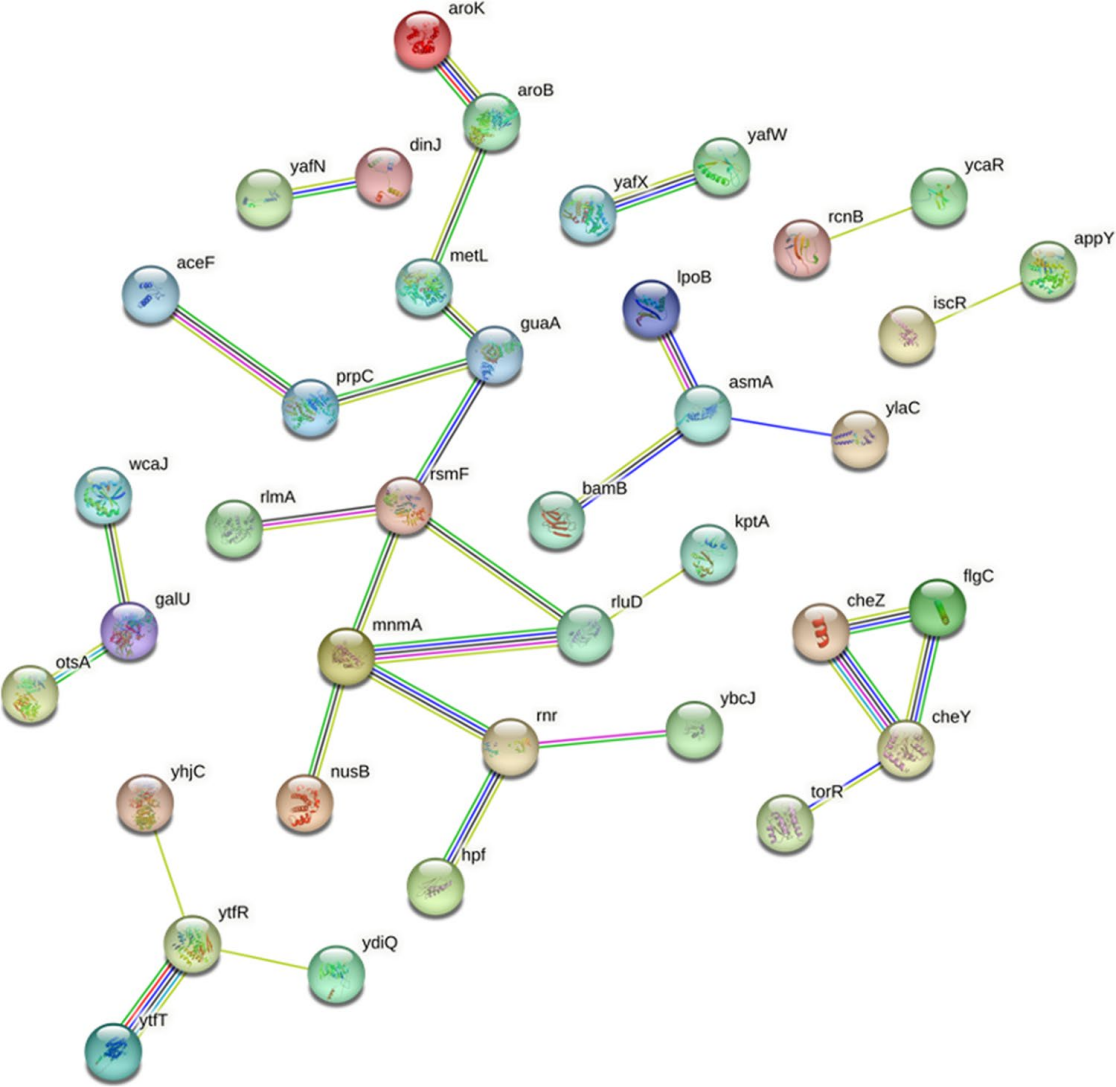
String analysis of the BA-sensitive gene hits. The predicted functional associations between the genes are shown by lines, where the number of the lines represents the linkage based on curated databases, gene-fusions, co-expression, and experimental evidence. STRING (version 10.5) with medium confidence score of 0.4 was used

Among the 10 virulence-related genes, the *dinJ* gene, YafQ-DinJ, is involved in the toxin-antitoxin system and encodes a polypeptide chain with DNA-binding transcriptional repressor activity. It is intriguing to find that a gene involved in the toxin-antitoxin system may be linked to intrinsic BA resistance. DinJ serves as the antitoxin component of the toxin-antitoxin (TA) module known as DinJ-YafQ. In this system, YafQ is a stable toxin, while DinJ is an unstable antitoxin. YafQ acts as a specific mRNA endoribonuclease that inhibits bacterial growth by impeding translation elongation under stress conditions. It has been shown to hinder protein synthesis, reduce growth rate, and inhibit colony growth. DinJ acts as an antitoxin that neutralizes YafQ’s activity by binding to it [75]. A study involving mutants of these two genes reported a decrease in biofilm formation [76]. Bacterial toxin-antitoxin (TA) systems have been associated with adaptation to stress conditions [77, 78]. *E. coli* harbors TA modules encoded by at least five gene pairs, namely, *relBE*, *mazEF*, *chpBIK*, *yefM*-*yoeB*, and *dinJ*-*yafQ* modules [78]. The DinJ-YafQ system has been confirmed as an active TA module [79]. The absence of the DinJ protein in a stressful environment has been shown to elevate RpoS levels, subsequently affecting the cell’s stress response [80]. The combination of *yafQ* and *dinJ* genes appears to be strongly linked, as shown by the STRING analysis (Fig. 5). The reason that the *dinJ* mutant is sensitive could be because of the increase in the activity of YafQ under BA stress. It may be a survival strategy for the bacterial population to keep some cells alive at the expense of sacrificing many others during environmental BA stress. However, further research is needed to confirm this interpretation, using various experimental approaches.

#### “Response to Stimulus”

*cheY* and *cheZ* genes are among the 19 genes classified as response to stimulus. The *cheZ* gene encodes the CheZ protein, which plays a role in chemotaxis. CheZ functions as a catalyst for the dephosphorylation of CheY, a response regulator involved in the chemotactic signaling pathway of *E. coli* [81, 82]. Chemotaxis refers to the positive or negative reaction of cells or free-moving organisms to a chemical stimulus.

One study examined motile, but often nonchemotactic (*che*) mutants of *E. coli*. The study evaluated six genes, two of which appear to belong to the “CheA” class (*cheA* and *cheW*), and four of which appear to belong to the “CheB” class (*cheX*, *cheB*, *cheY*, and *cheZ*). Mutations in the *cheA*, *cheW*, *cheX*, and *cheY* genes did not result in any functional impairment, while mutations in the *cheB* and *cheZ* genes led to a high rate of reduced mobility. These results indicate that these two processes are likely involved in regulating variations in flagellar movement in response to chemotactic stimuli [83]. In addition, the *flgC* gene encodes FlgC protein, one of the four proteins composing the flagellar basal body [84]. This complex is involved in interactions with components that determine the direction of the motor, as well as with CheY and CheZ chemotaxis proteins [85]. The STRING analysis in Fig. 5 reveals a well-connected network among the *cheZ*, *cheY*, and *flgC* genes, suggesting that the intrinsic resistance of *E. coli* to BA may be somehow related to its response to stimuli.

#### “Biyosynthesis”

Within the biosynthesis classification, there are 12 genes, including *aroK*, *aroB*, *metL*, and *guaA* genes. Additionally, the *wcaJ*, *galU*, and *otsA* genes are well linked, as shown by the STRING analysis in Fig. 5. The shikimate kinase and chorismate pathway play a role in producing aromatic amino acids such as tryptophan, tyrosine, and phenylalanine. AroK has been shown to possess shikimate kinase activity [86–88]. In a study, the A133P mutant of AroK was found to be linked to resistance to the mecillinam antibiotic [89]. Additionally, the *aroK* deletion mutant was found to be more sensitive to protamine, a cationic antibiotic peptide (CAMP). The researchers also suggested that AroK enhances the production of aromatic metabolites, which function as signaling molecules [90]. The *aroK* gene has also been associated with sensitivity to methyl methanesulfonate [91]. According to a study conducted in biofilm and planktonic growth media, the *aroK* gene was found to be important for survival in competitive planktonic growth conditions [92]. Our study reveals that *aroK* mutants are sensitive to BA.

Furthermore, in the study by Uluisik et al. (2011), it was demonstrated that boric acid stress affects the amino acid control mechanism in yeast through eIF2a phosphorylation, which is dependent on Gcn2 kinase [93]. These findings indicate that BA has an impact on amino acid metabolism, which is also supported by the results of our study.

## Conclusion

The Keio mutant line is a collection that enables the study of the phenotypes of thousands of non-essential gene mutants of *E. coli* in different environments. Our study conducted a genome-wide screening of 3985 *E. coli* mutants under varying concentrations of BA in the media, using the Keio collection to directly target the genes involved in intrinsic BA resistance. Bioinformatic analyses were conducted using the 92 BA-sensitive gene hits to identify processes and pathways associated with intrinsic BA resistance. The results showed that the “Regulation,” “Cell exterior,” and “Central Dogma” systems were relatively more affected by BA exposure. Notably, the functions of exoribonuclease II, RNA metabolic processes, and the regulation of chemotaxis activities were highlighted. In conclusion, our study indicates that intrinsic BA resistance in *E. coli* is achieved through multiple mechanisms working together within the cell. These findings offer valuable insights that can be used to generate new hypotheses for the scientific community investigating the mechanisms of BA on cellular life.

## Data Availability

The datasets generated and/or analyzed during the current study are available from the corresponding author on reasonable request.
